# Imported strongyloidiasis: Data from 1245 cases registered in the +REDIVI Spanish Collaborative Network (2009-2017)

**DOI:** 10.1371/journal.pntd.0007399

**Published:** 2019-05-16

**Authors:** Fernando Salvador, Begoña Treviño, Sandra Chamorro-Tojeiro, Adrián Sánchez-Montalvá, Juan María Herrero-Martínez, Azucena Rodríguez-Guardado, Núria Serre-Delcor, Diego Torrús, Josune Goikoetxea, Zuriñe Zubero, María Velasco, Elena Sulleiro, Israel Molina, Rogelio López-Vélez, José Antonio Pérez-Molina, on behalf the +REDIVI Collaborative Network

**Affiliations:** 1 Vall d’Hebron University Hospital, Universitat Autònoma de Barcelona, PROSICS Barcelona, Barcelona, Spain; 2 Tropical Medicine and International Health Unit Drassanes-Vall d’Hebron, PROSICS Barcelona, Barcelona, Spain; 3 Ramón y Cajal University Hospital, Madrid, Spain; 4 Hospital Universitario 12 de Octubre, Madrid, Spain; 5 Hospital Universitario Central de Asturias, Oviedo, Spain; 6 Hospital General Universitario de Alicante, Alicante, Spain; 7 Hospital Universitario Cruces, Barakaldo, Spain; 8 Hospital de Basurto, Bilbao, Spain; 9 Hospital Universitario Fundación Alcorcón, Madrid, Spain; Sacro Cuore Hospital, ITALY

## Abstract

**Background:**

Imported strongyloidiasis is increasingly being diagnosed in non-endemic areas. The aim of this study was to describe the epidemiological, clinical and microbiological characteristics of patients with imported strongyloidiasis in Spain.

**Methodology:**

This is an observational retrospective study that included all patients diagnosed of strongyloidiasis registered in the +REDIVI Collaborative Network from 2009 to 2017. Demographic, epidemiological and clinical information was collected from the +REDIVI database, and extra information regarding microbiological techniques, treatment and follow-up was requested to participant centers.

**Findings:**

Overall, 1245 cases were included. Most of them were immigrants (66.9%), and South America was the most frequent area of origin. Detection of larvae in stool samples was observed in 21.9% of the patients, and serological tests allowed making the diagnosis in the rest of the cases. Eosinophilia was present in 82.2% of cases. Treatment with ivermectin (compared with albendazole) was the most strongly associated factor to achieve the cure (OR 2.34).

**Conclusions:**

Given the long latency of the infection and the risk of developing a severe presentation, screening of *S*. *stercoralis* infection should be mandatory in patients coming from or had traveling to endemic areas, especially in those with immunosuppressant conditions.

## Author summary

Strongyloidiasis, the parasitic disease caused by the nematode *Strongyloides stercoralis*, is being increasingly diagnosed in immigrant population in Spain. In the present study we describe de clinical, epidemiological and microbiological profile of 1245 cases of imported strongyloidiasis registered in the +REDIVI Spanish Collaborative Network. Patients were mostly immigrants, and the majority of them were coming from South America. Serological tests allowed the diagnosis in most of the cases, and detection of larvae in stool samples was associated with male gender and presence of eosinophilia. Ivermectin was associated with higher probability of cure compared with albendazole. Given the risk of developing a severe presentation, screening of *S*. *stercoralis* infection should be mandatory in patients coming from or had traveling to endemic areas, especially in those with immunosuppressant conditions.

## Introduction

Strongyloidiasis is caused by the soil-transmitted nematode *Strongyloides stercoralis* [1]. The parasite is globally distributed in the tropics and occurs in some parts of the sub-tropics, although it has also been reported in more temperate climates, like southern Europe and North America [2]. It has been recently estimated that 370 million people are infected by the parasite; however, this prevalence is most probably underestimated, since the majority of the studies are focused on other soil-transmitted helminthes, and the microbiological techniques commonly used have low sensitivity for *S*. *stercoralis* [3, 4].

*S*. *stercoralis* infection is most often asymptomatic, but patients may present with gastrointestinal, cutaneous, and respiratory symptoms [2]. This parasite has the unique ability to persist in the human host for decades without the need of exogenous reinfection due to its autoinfection life cycle. Nevertheless, some immunosuppressant conditions, such as corticosteroid therapy, solid organ or bone marrow transplantation and Human T-lymphotropic virus 1 (HTLV-1) infection, may amplify this autoinfective cycle, leading to severe presentations such as the *S*. *stercoralis* hyperinfection syndrome and the disseminated strongyloidiasis [5, 6].

Due to increase of migrant flows and international travels during the last decades, imported strongyloidiasis is being increasingly diagnosed in Spain, as well as in other non-endemic areas, becoming a worldwide public health challenge [7–11]. The aim of the present study was to describe the epidemiological, clinical and microbiological characteristics of patients with imported strongyloidiasis registered in the +REDIVI Spanish Collaborative Network, as well as to identify potential factors related to parasite burden and treatment success.

## Material and methods

### Study design and study population

This is an observational retrospective study that included all patients diagnosed of strongyloidiasis registered in the +REDIVI Collaborative Network from January 2009 to February 2017. This Spanish network includes 22 centers which share a common online database to register epidemiological and clinical information of immigrants and travelers who attend these centers. The database includes: demographic information (date of birth, gender, and country of birth), epidemiological data (country of origin for immigrants, travel characteristics), and clinical information (main reason for consultation, and diagnosis). +REDIVI includes 4 types of patients: immigrants (people living in Spain and born in other country), Visiting Friends and Relatives (VFR)-immigrants (immigrants who have travelled to their country of birth to visit friends and relatives), VFR-travelers (people born in Spain who have travelled to their first-degree relative’s country of birth), and travelers (people born in Spain who return from international travels).

All participant centers were requested to contribute with extra information from the included patients: HTLV-1 co-infection, eosinophil cell count at diagnosis, microbiological techniques performed and their results, treatment information, and follow-up controls up to two visits.

### Microbiological techniques

Stool microscopic examination was performed using the Ritchie’s formalin-ether technique in all centers. The specific fecal culture for *S*. *stercoralis* larvae was performed using the agar plate or charcoal technique. Detection of serum anti-*S*. *stercoralis* IgG was performed through different enzyme-linked immunosorbent assay (ELISA)-based commercial kits depending on the center: SciMedx *Strongyloides* serology microwell ELISA (SciMedx Corporation, Denville, NJ, United States), NovaLisa Strongyloides (NovaTec Immunodiagnostica GmbH, Dietzenbach, Germany), *Strongyloides* IgG IVD-ELISA (DRG Instruments GmbH, Marburg, Germany), AccuDiag *Strongyloides* IgG ELISA Kit (Diagnostic Automotion/Cortez Diagnostics Inc, CA, United States). All serological tests were performed and cut-off of the optical density values to determine a positive result was established following manufacturer instructions.

### Definitions

Eosinophilia was defined as eosinophil cell count ≥ 0.45x10^9^cells/L and/or a percentage ≥5%: mild (<1.0x10^9^cells/L), moderate (1.0–3.0x10^9^cells/L), and severe (>3.0x10^9^cells/L). The *S*. *stercoralis* infection diagnosis was classified into three groups: definite (detection of larvae by any parasitological technique), probable (positive serological result and presence of eosinophilia), and possible (positive serological result without presence of eosinophilia). We considered severe clinical presentation: hyperinfection syndrome (infection confined to lungs and gastrointestinal tract, but symptoms of severe disease related to elevated number of larvae) and disseminated strongyloidiasis (larvae found in any organ other than the lungs and gastrointestinal tract). In order to evaluate the treatment outcome, four categories were considered. The “cure” criteria was established when patients had negative conventional methods (microscopic stool examination and/or fecal culture) after treatment, disappearance of the eosinophilia, and negative serology (or at least a 60% decrease in the OD ratio) during follow-up (independently of the time they reached this situation). We established “probable cure” criteria when the cure criteria were reached but eosinophilia persisted (because it could be to other cause) or when in the absence of serologic control, patients had negative conventional methods after treatment and disappearance of the eosinophilia. “Failure” was considered if larvae were detected after the treatment or when the cure or probable cure criteria regarding serology were not reached during follow-up (with a minimum of 6 months of follow-up). The outcome “not enough information” was considered when the patients had negative conventional methods after treatment, but cure or probable cure criteria regarding eosinophilia and serology were not reached during follow-up (with a follow-up period less than 6 months).

### Statistical analysis and ethical issues

The STROBE statement guidelines were used to improve the quality of the study [12]. Procedures were performed in accordance with the ethical standards laid down in the Declaration of Helsinki as revised in 2013, and the study protocol was approved by the Ethical Review Board of the Vall d’Hebron University Hospital (Barcelona, Spain). The Ethical Review Board was consulted, and agreed that informed consent was not necessary given de retrospective nature of the study and the anonymization of the information.

Categorical data are presented as absolute numbers and proportions, and continuous variables are expressed as means and standard deviations (SD) or medians and interquartile ranges (IQR) depending on the distribution. The Kolmogorov-Smirnov test was used to evaluate the normal distribution of variables. The χ^2^ test or Fisher exact test, when appropriate, was used to compare the distribution of categorical variables, and the t-Student test for continuous variables. Multivariate logistic regression analysis was made to identify factors associated with confirmed diagnosis and cure. Variables were entered in the model if p<0.20 on univariate analysis. Results were considered statistically significant if the 2-tailed P value was <0.05. SPSS software for Windows (Version 19.0; SPSS Inc, Chicago, IL, USA) was used for statistical analyses.

## Results

**Fig 1:**
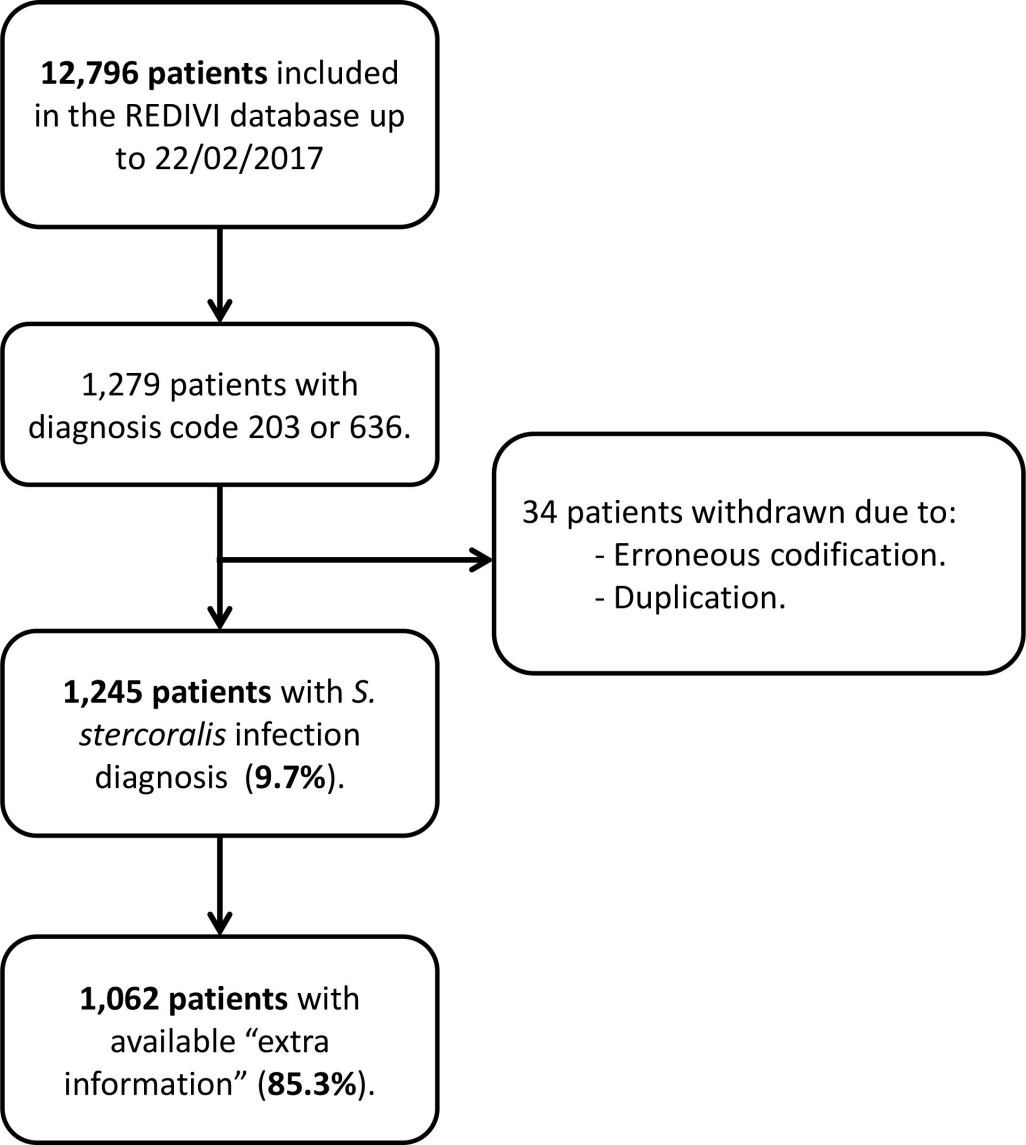
Flow diagram of patients.

Overall, 12796 patients were registered in +REDIVI from January 2009 to February 2017. Of these, 1279 patients had the *S*. *stercoralis* infection diagnosis. After revising these cases, 34 patients were withdrawn due to erroneous codification or duplication; hence, resulting in 1245 (9.7%) patients with *S*. *stercoralis* infection diagnosis (Fig 1). The mean age at the time of diagnosis was 38.3 (SD 12.9) years, and 590 (47.4%) were men (see Table 1). Most of them were immigrants (833 patients, 66.9%), and 80 (6.4%) patients had some kind of immunosuppressant condition. The main reasons for consultation were “laboratory test alteration” (41.1%) and “health screening” (39.4%). Only 5 (0.4%) patients had the diagnosis of hyperinfection syndrome: two patients with autoimmune diseases who were receiving immunosuppressive therapies (including corticosteroids), one patient with HTLV-1 infection, one patient with HIV infection, and one patient without any immunosuppressant condition. Six hundred and five (48.6%) patients had at least another infectious disease at the time of strongyloidiasis diagnosis, being Chagas disease (21.8%), latent tuberculosis infection (6%), and schistosomiasis (5%) the most prevalent ones (S1 Table).

**Table 1:** Clinical and demographic characteristics of patients with strongyloidiasis in +REDIVI (2009–2017).

Clinical and demographic characteristics	Number of patients (n = 1245)
**Demographic information**	
Age, years	38.3 (SD 12.9, 2–88)
Gender, male	590 (47.4%)
**Epidemiological groups**	
Immigrants	833 (66.9%)
VFR-immigrants	338 (27.1%)
Travelers	74 (6%)
**Immunosuppressant conditions**	
None	1165 (93.6%)
HIV infection	55 (4.4%)
Immunosuppressive drugs	15 (1.2%)
Organ transplantation	5 (0.4%)
Other	5 (0.4%)
**Main reason for consultation**	
Laboratory test alteration	512 (41.1%)
Health screening	491 (39.4%)
Gastrointestinal symptoms	105 (8.4%)
Cutaneous manifestations	105 (8.4%)
Fever	33 (2.7%)
Respiratory symptoms	9 (0.7%)
Bone/muscle manifestations	8 (0.6%)
Cardiovascular symptoms	7 (0.6%)
Lymphadenopathy	4 (0.3%)
Other	17 (1.4%)
**Clinical presentation**	
Non-complicated strongyloidiasis	1240 (99.6%)
Hyperinfection syndrome	5 (0.4%)

**NOTE.** Data are reported as number (%) of patients or mean (SD, range)

Among the 833 immigrants, South America was the most frequent geographical area of origin, and mean time of residence in Spain was 6.8 (SD 5.4) years. The median duration of the trip in travelers and VFR-immigrants were 45 (IQR 19.7–227) days and 30 (IQR 30–60) days respectively, and Sub-Saharan Africa and South America were the most frequent geographical areas of travel destination (S2 Table).

After requesting extra information of the included patients to the participant centers, it was available from 1062 (85.3%) patients. Regarding *S*. *stercoralis* infection diagnosis, patients could be classified as follows: definite in 233 (21.9%) patients, probable in 648 (61%) patients, and possible in 181 (17.1%) patients. Proportion of positive results for each microbiological technique was: 17.4% for stool formalin-ethyl acetate sedimentation, 22.5% for *S*. *stercoralis* fecal culture, and 98.8% for *S*. *stercoralis* serology. These differences were higher when analyzing the efficiency of the microbiological techniques in the 561 patients in whom all three techniques were performed (see Table 2). Nine hundred and sixty-eight patients received specific treatment for strongyloidiasis; 94 patients did not return to the centers after the diagnosis, and treatment could not be offered to them. Ivermectin was the drug of choice in 90.4% of the cases. From the 968 patients who received treatment, 784 (81%) had at least one follow-up control, with median time of follow-up of 6 (IQR 3–11) months. Treatment outcomes of these patients were as follows: 301 (38.4%) cure, 350 (44.6%) probable cure, 54 (6.9%) treatment failure, and 79 (10.1%) with no enough information. From the 54 patients considered treatment failure, only in four cases was due to larvae detection after treatment: a 40-years-old man coming from Sudan (treated with 200mcg/day x 2days, larvae detected after 4 months), a 28-years-old women coming from Bolivia (treated with 200mcg/day x 2days, larvae detected after 6 months), a 35-years-old women coming from Equatorial Guinea (with HIV infection, treated with 200mcg/day x 2days, larvae detected after 3 months), and a 43-years-old man coming from Bolivia (treated with albendazole 400mg/12 hours x 7 days, larvae detected after 3 months). Therefore, 83% of the patients achieved a successful outcome (cure or probable cure). More information regarding the treatment and follow-up results is summarized in Table 3 and S3 Table.

**Table 2:** Laboratorial and microbiological information at the time of diagnosis of patients with strongyloidiasis in +REDIVI with available extra information (2009–2017).

Laboratory and microbiology information of all patients	Number of patients (n = 1062)	Laboratory and microbiology information of patients in whom all three microbiological techniques were performed	Number of patients (n = 561)
**Presence of eosinophilia**	867/1055 (82.2%)	**Positive microbiological techniques**	
Mild (<1.0x10^9^cells/L)	490/867 (56.5%)	Stool microscopic investigation	52/561 (9.3%)
Moderate (1.0–3.0x10^9^cells/L)	356/867 (41.1%)	*S*. *stercoralis* specific faecal culture	84/561 (15%)
Severe (>3.0x10^9^cells/L)	21/867 (2.4%)	*S*. *stercoralis* serology	554/561 (98.8%)
**HTLV-1 infection**	9 /224 (4%)	**Patients with positive results in all techniques**	40/561 (7.1%)
***S*. *stercoralis* infection diagnosis**		**Patients with 2 positive results**	49/561 (8.8%)
Definite	233/1062 (21.9%)	Serology + culture	37/561 (6.6%)
Probable	648/1062 (61%)	Serology + microscopic investigation	8/561 (1.5%)
Possible	181/1062 (17.1%)	Culture + microscopic investigation	4/561 (0.7%)
**Positive microbiological techniques**		**Patients with only 1 positive result**	472/561 (84.1%)
Stool microscopic investigation	173/995 (17.4%)	Serology	469/561 (83.6%)
*S*. *stercoralis* specific faecal culture	145/643 (22.5%)	Culture	3/561 (0.5%)
*S*. *stercoralis* serology	935/946 (98.8%)	Microscopic investigation	0/561 (0%)

**NOTE.** Data are reported as number (%) of patients.

**Table 3:** Treatment and follow-up information of patients with strongyloidiasis in +REDIVI with available extra information (2009–2017).

Drug of choice (n = 968)		Duration of the treatment		Follow-up (n = 968)	
**Ivermectin**	875 (90.4%)	**Duration for ivermectin (n = 884)**		**Number of follow-up controls**	
150mcg/Kg/day po	94 (9.7%)	1 day	31 (3.5%)	No control	184 (19%)
200mcg/Kg/day po	780 (80.6%)	2 days	840 (95%)	One control	520 (53.7%)
200mcg/Kg/day po+sc	1 (0.1%)	4 days	9 (1%)	Two controls	264 (27.3%)
**Albendazole 400mg/12 hours po**	84 (8.7%)	7 days	3 (0.4%)	**Mean time of follow-up, months**	6 (IQR 3–11)
**Ivermectin 200mcg/Kg/day po + albendazole 400mg/12 hours**	9 (0.9%)	20 days	1 (0.1%)	**Treatment outcome (n = 784)**	
		**Duration for albendazole (n = 93)**		Cure	301 (38.4%)
		3 day	5 (5.4%)	Probable cure	350 (44.6%)
		5 days	34 (36.6%)	Treatment failure	54 (6.9%)
		6 days	1 (1.1%)	No enough information	79 (10.1%)
		7 days	48 (51.6%)		
		8 days	1 (1.1%)		
		10 days	2 (2.1%)		
		15 days	2 (2.1%)		

**NOTE.** Data are reported as number (%) of patients or median (IQR). Po, peroral; sc, subcutaneous.

When analyzing potential factors associated with having a definite diagnosis, after the multivariate analysis, the presence of eosinophilia was the most strongly associated factor, with an OR of 3.83 (95%CI 2.09–7.04). Presence of moderate/severe eosinophilia and male gender were also associated with confirmed diagnosis, with an OR of 1.57 (95%CI 1.15–2.14) and 1.69 (95%CI 1.25–2.29) respectively. Conversely, the immigrant group (compared with VFR-immigrants and travelers) had a negative association with definite diagnosis, with an OR of 0.64 (95%CI 0.47–0.87). More information is depicted in Table 4. Regarding factors associated to achieving the cure criteria, treatment with ivermectin (compared with treatment with albendazole) was the most strongly associated factor, with an OR of 2.34 (95%CI 1.23–4.47). Male gender, presence of eosinophilia, and definite diagnosis had a negative association for achieving the cure criteria (see Table 5).

**Table 4:** Factors associated to definite diagnosis in patients with strongyloidiasis in +REDIVI with available extra information (2009–2017).

	Overall (n = 1062)	Definite diagnosis (n = 233)	Probable diagnosis (n = 829)	Univariate analysis (*P* value)	Multivariate analysis (OR, 95%CI, and *P* value)
Mean age at diagnosis, years	37.9 (SD 13.04, 2–88)	39.4 (SD 12.7, 2–88)	37.5 (SD 13.1, 2–82)	0.058	OR 1.01 (0.99–1.02), p = 0.181
Gender, male	520/1062 (49%)	136/233 (58.4%)	384/829 (46.3%)	0.001	**OR 1.69 (1.25–2.29), p = 0.001**
Immigrants^a^	699/1062 (65.8%)	136/233 (58.4%)	563/829 (67.9%)	0.007	**OR 0.64 (0.47–0.87), p = 0.006**
Time of residence in Spain, years (n = 699)^b^	6.4 (SD 5.1, 0–34)	5.6 (SD 4.3, 0–21)	6.5 (SD 5.2, 0–34)	0.050	-
Presence of immunosuppressant condition	71/1062 (6.7%)	13/233 (5.6%)	58/829 (7%)	0.444	-
HTLV-1 co-infection (n = 224)	9/224 (4%)	3/55 (5.5%)	6/169 (3.6%)	0.692	-
Presence of eosinophilia (n = 1055)	867/1055 (82.2%)	219/232 (94.4%)	648/823 (78.7%)	<0.001	**OR 3.83 (2.09–7.04), p<0.001**
Moderate/Severe eosinophilia (n = 1055)	377/1055 (35.7%)	113/232 (48.5%)	264/823 (32.1%)	<0.001	**OR 1.57 (1.15–2.14), p = 0.004**
Presence of symptoms^c^	190/1062 (17.9%)	40/233 (17.2%)	150/829 (18.1%)	0.744	-

**NOTE.** Data are reported as number (%) of patients or mean (SD, range).

^a^ Immigrant group compared to VFR-immigrants and travelers.

^b^ Time of residence in Spain in immigrant patients.

^c^ Patients in whom the main reason for consultation were not laboratory test alteration nor health screening.

**Table 5:** Factors associated to cure in patients with strongyloidiasis in +REDIVI with available extra information (2009–2017).

	Overall (n = 705)^a^	Cure (n = 301)	Not cure (n = 404)	Univariate analysis (*P* value)	Multivariate analysis (OR, 95%CI, and *P* value)
Mean age at diagnosis, years	39.8 (SD 12.8, 2–88)	40 (SD 12.5, 5–71)	39.2 (SD 13.1, 2–88)	0.132	OR 1.01 (0.99–1.02), p = 0.132
Gender, male	344/705 (48.8%)	130/301 (43.2%)	214/404 (53%)	0.010	**OR 0.64 (0.47–0.88), p = 0.007**
Presence of immunosuppressant condition	50/705 (7.1%)	27/301 (9%)	23/404 (5.7%)	0.094	OR 1.37 (0.73–2.56), p = 0.314
HTLV-1 co-infection (n = 152)	7/152 (4.6%)	4/105 (3.8%)	3/47 (6.4%)	0.677	-
Presence of eosinophilia (n = 704)	608/704 (86.4%)	241/301 (80.1%)	367/403 (91.1%)	<0.001	**OR 0.49 (0.30–0.78), p = 0.003**
Moderate/Severe eosinophilia (n = 704)	268/704 (38.1%)	107/301 (35.5%)	161/403 (39.9%)	0.234	-
Definite diagnosis	178/705 (25.2%)	50/301 (16.6%)	128/404 (31.7%)	<0.001	**OR 0.44 (0.30–0.64), p<0.001**
Presence of symptoms^b^	122/705 (17.3%)	63/301 (20.9%)	59/404 (14.6%)	0.028	OR 1.42 (0.73–2.56), p = 0.094
Treatment with ivermectin (n = 698) ^c^	644/698 (92.3%)	285/299 (95.3%)	359/399 (90%)	0.009	**OR 2.34 (1.23–4.47), p = 0.009**

**NOTE.** Data are reported as number (%) of patients or mean (SD, range).

^a^ Patients with not enough information to establish the treatment outcome were withdrawn from the analysis.

^b^ Patients in whom the main reason for consultation were not laboratory test alteration nor health screening.

^c^ Patients were only analyzed when treated with a single drug (ivermectin or albendazole).

## Discussion

Imported strongyloidiasis is increasingly being diagnosed in non-endemic countries, especially in Spain, who receives an important number of immigrants coming from endemic areas, although information comes from short series of cases [7–11]. Here we present a cohort of 1245 patients with imported strongyloidiasis in Spain: most of them were asymptomatic immigrants coming from South America, 82.2% had eosinophilia at the time of diagnosis, 6.4% of them had some kind of immunosuppressant condition, almost half of them had another infectious disease (being Chagas disease the most frequent one), serology allowed the diagnosis in most of the cases, and treatment with ivermectin was associated with better outcome.

It is important to note that, although the percentage of patients with eosinophilia was very high (more than 80%), the eosinophilia could be due to other causes, since there were patients co-infected with other helminthiases such as schistosomiasis, soil-transmitted helminths, or filarial nematodes. Hence, the percentage of patients with eosinophilia could be overestimated.

As in other imported strongyloidiasis series, most of the cases were diagnosed in immigrants and VFR-immigrants [7–11]. It is important to note that strongyloidiasis in VFR may have been acquired prior to residence in Spain rather than during the recent travel to their country of origin. Strongyloidiasis in travelers, although described, is much more infrequent, and restricted to high risk travelers, such as long-term travelers, humanitarian workers, and backpackers. In a study performed in The Netherlands where 679 asymptomatic long-term travelers to the tropics (median travel duration of 12 weeks) were post-travel screened for intestinal parasites using molecular techniques, only one (0.1%) traveler was diagnosed of strongyloidiasis [13]. In our study, travelers represented 6% of the total study population, which is higher than in other studies; it could be due to the epidemiological profile of travelers registered in +REDIVI, with high percentage of high risk travelers (missionaries and humanitarian workers). Among immigrants and VFR-immigrants, South America was the most frequent geographical area of origin, which is concordant with the general immigrant population in Spain, probably due to cultural and historic reasons. Spanish Tropical Medicine Units are highly sensitized in offering Chagas disease screening in South American population, especially those coming from Bolivia. This is the reason of the high proportion of patients coming from Bolivia in our study, and the high proportion of Chagas disease-strongyloidiasis co-infection. This co-infection has been recently found to increase the likelihood to detect *Trypanosoma cruzi* DNA in peripheral blood, which may reflect the potential immunomodulatory effects of *S*. *stercoralis* infection [14].

The percentage of severe clinical manifestations in our study (0.4%) could be underestimated, since most of the patients were referred to the centers for screening their health status or eosinophilia investigation. From the five patients with hyperinfection syndrome, four had some immunosuppressant risk factor (80%), whereas only 6% of patients without hyperinfection syndrome were immunosuppressed. Two of them were receiving corticosteroids, which was the most related risk factor found in a recent systematic review that included 244 cases of severe strongyloidiasis [6]. HTLV-1 infection increases the risk of *S*.*stercoralis* infection, the risk of developing a severe strongyloidiasis, and the risk of treatment failure [15]. In our study, 9 patients had a positive HTLV-1 serological test (one of them presenting a severe strongyloidiasis), although the test was only performed in 224 patients (21% of the study population).

There is currently no gold standard for *S*. *stercoralis* infection diagnosis, since the available techniques are not sensitive enough. Larvae detection in stool or other samples gives a definite diagnosis, but the scarce and irregular larvae excretion in chronically infected patients makes the diagnosis challenging. Although parasitological techniques have been improving (concentration techniques, Harada-Mori, Baermann, fecal culture), these techniques are not sensitive enough [16, 17]; in our study, the proportion of positive results in patients in whom all three techniques were performed was 9.3% for the formalin-ether technique and 15% for the fecal culture. The presence of eosinophilia was strongly associated with higher proportion of definite diagnosis, which may reflect a higher larvae excretion, possibly related to a higher parasite burden in these patients. Male gender was also associated with definite diagnosis. Both factors have been previously associated with *Strongyloides* infection in studies where the infection was defined by positive parasitological techniques [18, 19]. Conversely, being immigrant had a negative association with definite diagnosis. It could be explained due to the chronic situation of the infection in this group of patients (they have leaved the endemic region years ago), and the fact that many of them have been diagnosed during a health screening, without presenting neither symptoms nor eosinophilia. In recent years, DNA detection-based techniques (such as PCR) have been developed, showing higher sensitivity than classical parasitological techniques [20].

Fortunately, different serological techniques have been developed during the last decades in order to increase the sensitivity in the strongyloidiasis diagnosis, being the ELISA-based techniques the ones with highest sensitivity [21]. Previous studies have shown the usefulness of *S*. *stercoralis* serology in the diagnosis of strongyloidiasis, especially in those presenting eosinophilia with negative coproparasitological examination [8, 22]. Serology allowed strongyloidiasis diagnosis in 78.1% of the patients in our study, becoming a key tool in the management of these patients. Moreover, serological tests are also useful to evaluate the treatment response, although a uniform criterion to define cure has not yet been established. In general, a negative serology or a 50–60% decrease in the optical density after treatment is considered a cure criteria or a successful outcome, which was the criteria used in the present study, although the time when this decrease should be determined is not well established [8, 23–25]. Nevertheless, *S*. *stercoralis* serology may overestimate the prevalence of strongyloidiasis, since it may remain positive after resolution of the infection or could cross-react with other helminth infections. It has been suggested that increasing the cut-off value for positive serology may increase the specificity of the serological test in the diagnosis of confirmed strongyloidiasis [26]. Other diagnostic strategy could include a combination of different techniques, such as classical parasitological methods, serology, and even PCR.

Some clinical trials have compared the efficacy between ivermectin and albendazole for the treatment of chronic strongyloidiasis, with a cure rate ranging from 76% to 98% and 38% to 78% respectively. All these clinical trials included a relative low number of subjects, used different treatment schemes and dosages, and assessed the treatment success using classical parasitological methods. As mentioned before, this strategy may have overestimated the cure response due to lack of sensitivity [27–32]. A recent Cochrane review has performed a meta-analysis comparing the efficacy of ivermectin versus albendazole including 478 participants, showing higher parasitological cure with ivermectin (RR 1.79, 95%CI 1.55–2.08) with moderate quality evidence [33]. Although our study had a retrospective design, a high number of patients (698 participants) could be evaluated to compare the efficacy of ivermectin versus albendazole; and more interestingly, our study included serological techniques during the follow-up, that increases the possibility to detect treatment failures. Even though the evidence of ivermectin superiority, almost 10% of the patients in our study were treated with albendazole; it could be explained because most of these cases took place at the beginning of the study (the evidence was less than nowadays), and the availability of ivermectin in some Spanish regions is limited. Patients with eosinophilia or with definite diagnosed were less likely to achieve the cure criteria; although it could reflect higher parasitic load in these patients, the retrospective nature of the study makes difficult the interpretation of these results. Male gender was also negatively associated to cure, which has been previously described in other studies [30].

The results of the previously mentioned clinical trials are very different to and contradict with the results of a recent observational study performed in a non-endemic area (Buenos Aires, Argentina). In this study, 21 patients with strongyloidiasis were treated with ivermectin; during follow-up, in 14 patients larvae were detected in stools by conventional methods, and *S*. *stercoralis* DNA was detected by PCR in all patients [34]. Although these results are surprising, they should be taken with caution, since come from a small observational study, and stronger evidence (coming from clinical trials) shows high efficacy of ivermectin. In our study, although 79 patients were considered treatment failure, only in 4 patients larvae were detected by conventional methods. Management of strongyloidiasis treatment failure is not standardized; the general attitude of the participant centres in our study in these situations was to repeat a course of ivermectin or to treat with a long course of albendazole.

This study has some limitations given its retrospective nature. Patients were diagnosed through different serological tests, and information regarding the number of stool samples tested in each patient was not collected, and it could influence in the sensitivity of fecal-based diagnostic techniques. Diagnostic tests and time of evaluation during follow-up were different depending on the centre. Moreover, the number of patients treated with albendazole was very low, and it difficult the interpretation of the efficacy comparison between the two drugs. Hence, the results have to be interpreted with caution. Despite these limitations, the present study has very strong points, such as the high number of included patients, the participation of 22 centers distributed through different Spanish regions, and reflects how Tropical Medicine specialists diagnose and manage patients with strongyloidiasis in a non-endemic setting under real conditions. The collaborative networks, such as the Spanish +REDIVI network, give the possibility to gather information from high number of patients, being very useful to study some rare manifestations from common diseases [35]. It will allow us to further investigate situations such as severe strongyloidiasis or strongyloidiasis in immunosuppressed patients.

In summary, we present a cohort of 1245 patients with imported strongyloidiasis in Spain. Patients were mostly immigrants, and the majority of them were coming from South America. Serological tests allowed the diagnosis in most of the cases, and detection of larvae in stool samples was associated with male gender and presence of eosinophilia. Ivermectin was associated with higher probability of cure compared with albendazole. Given the risk of developing a severe presentation, screening of *S*. *stercoralis* infection should be mandatory in patients coming from or had traveling to endemic areas, especially in those with immunosuppressant conditions [36]. Although parasitological techniques are the only that can confirm the diagnosis, to include serological techniques is very useful both at diagnosis and follow-up. Whenever possible, ivermectin must be the drug of choice. And finally, given the high number of immigrants and travelers coming from endemic areas that live in Spain, although unlikely under current hygienic conditions, reintroduction of the disease in our country could potentially occur, which would have important public health implications.

## Supporting information

S1 Checklist STROBE checklist. (DOCX)

S1 Table Supplementary Tables S1. (DOC)

S2 Table Suplementary Table S2. (DOC)

S3 Table Suplementary Table S3. (DOC)

S1 Appendix Members of the +REDIVI study group. (DOC)

## References

[1] World Health Organization. Strongyloidiasis. Data available on www.who.int/neglected_diseases/diseases/strongyloidiasis/en/. Last accessed on December 2018.

[2] Puthiyakunnon S, Boddu S, Li Y, Zhou X, Wang C, Li J et al Strongyloidiasis—an insight into its global prevalence and management. PLoS Negl Trop Dis. 2014; 8: e3018 10.1371/journal.pntd.0003018 25121962 PMC4133206

[3] Schär F, Trostdorf U, Giardina F, Khieu V, Muth S, Marti H et al Strongyloides stercoralis: global distribution and risk factors. PLoS Negl Trop Dis. 2013; 7: e2288 10.1371/journal.pntd.0002288 23875033 PMC3708837

[4] Olsen A, Van Lieshout L, Marti H, Polderman T, Polman K, Steinmann P et al. Strongyloidiasis—the most neglected of the neglected tropical diseases? Trans R Soc Trop Med Hyg. 2009; 103: 967–972. 10.1016/j.trstmh.2009.02.013 19328508

[5] Keiser PB, Nutman TB. *Strongyloides stercoralis* in the immunocompromised population. Clinical Microbiology Reviews, 2004; 17: 208–217. 10.1128/CMR.17.1.208-217.2004 14726461 PMC321465

[6] Buonfrate D, Requena-Mendez A, Angheben A, Muñoz J, Gobbi F, Van den Ende J et al Severe strongyloidiasis: a systematic review of case reports. BMC Infect Dis. 2013; 13: 78 10.1186/1471-2334-13-78 23394259 PMC3598958

[7] Buonfrate D, Baldissera M, Abrescia F, Bassetti M, Caramaschi G, Giobbia M et al Epidemiology of Strongyloides stercoralis in northern Italy: results of a multicentre case-control study, February 2013 to July 2014. Euro Surveill. 2016; 21: 30310. 10.2807/1560-7917.ES.2016.21.31.30310 PMC4998510 27525375

[8] Salvador F, Sulleiro E, Sánchez-Montalvá A, Saugar JM, Rodríguez E, Pahissa A et al Usefulness of *Strongyloides stercoralis* serology in the management of patients with eosinophilia. Am J Trop Med Hyg. 2014; 90: 830–834. 10.4269/ajtmh.13-0678 24615124 PMC4015573

[9] Ramírez-Olivencia G, Espinosa MA, Martín AB, Núñez NI, De las Parras ER, Núñez ML et al Imported strongyloidiasis in Spain. Int J Infect Dis. 2014; 18: 32–37. 10.1016/j.ijid.2013.09.009 24211226

[10] Ostera G, Blum J, Cornejo C, Burgula S, Jeun R, Bryan PE et al Strongyloidiasis in Latin American immigrants: a pilot study. J Helminthol. 2017; 91: 262–266. 10.1017/S0022149X16000213 27121364

[11] Cabezas-Fernández MT, Salas-Coronas J, Lozano-Serrano AB, Vázquez-Villegas J, Cabeza-Barrera MI, Cobo F. Strongyloidiasis in immigrants in Southern Spain. Enferm Infecc Microbiol Clin. 2015; 33: 37–39. 10.1016/j.eimc.2014.06.010 25205127

[12] Vandenbroucke JP, Von Elm E, Altman DG, Gotzsche PC, Mulrow CD, Pocock SJ et al Strengthening the Reporting of Observational Studies in Epidemiology (STROBE): explanation and elaboration. Int J Surg. 2014; 12: 1500–1524. 10.1016/j.ijsu.2014.07.014 25046751

[13] Soonawala D, Van Lieshout L, Den Boer MA, Claas EC, Verweij JJ, Godkewitsch A et al Post-travel screening of asymptomatic long-term travelers to the tropics for intestinal parasites using molecular diagnostics. Am J Trop Med Hyg. 2014; 90: 835–839. 10.4269/ajtmh.13-0594 24615130 PMC4015574

[14] Salvador F, Sulleiro E, Piron M, Sánchez-Montalvá A, Sauleda S, Molina-Morant D et al *Strongyloides stercoralis* infection increases the likelihood to detect *Trypanosoma cruzi* DNA in peripheral blood in Chagas disease patients. Trop Med Int Health. 2017; 22: 1436–1441. 10.1111/tmi.12970 28869694

[15] Carvalho EM, Da Fonseca Porto A. Epidemiological and clinical interaction between HTLV-1 and *Strongyloides stercoralis*. Parasite Immunol. 2004; 26: 487–497. 10.1111/j.0141-9838.2004.00726.x 15771684

[16] Intapan PM, Maleewong W, Wongsaroj T, Singthong S, Morakote N. Comparison of the quantitative formalin ethyl acetate concentration technique and agar plate culture for diagnosis of human strongyloidiasis. J Clin Microbiol. 2005; 43: 1932–1933. 10.1128/JCM.43.4.1932-1933.2005 15815023 PMC1081356

[17] Kaminsky RG. Evaluation of three methods for laboratory diagnosis of *Strongyloides stercoralis* infection. J Parasitol. 1993; 79: 277–280. 8459339

[18] Jongwutiwes U, Waywa D, Silpasakorn S, Wanachiwanawin D, Suputtamongkol Y. Prevalence and risk factors of acquiring Strongyloides stercoralis infection among patients attending a tertiary hospital in Thailand. Pathog Glob Health. 2014; 108: 137–140. 10.1179/2047773214Y.0000000134 24766337 PMC4083175

[19] Chordia P, Christopher S, Abraham OC, Muliyil J, Kang G, Ajjampur SSR. Risk factors for acquiring *Strongyloides stercoralis* infection among patients attending a tertiary hospital in south India. Indian J Med Microbiol. 2011; 29: 147–151. 10.4103/0255-0857.81797 21654109

[20] Dacal E, Saugar JM, Soler T, Azcárate JM, Jiménez MS, Merino FJ, Rodríguez E. Parasitological versus molecular diagnosis of strongyloidiasis in serial stool samples: how many? J Helminthol. 2018; 92: 12–16. 10.1017/S0022149X17000050 28112060

[21] Requena-Méndez A, Chiodini P, Bisoffi Z, Buonfrate D, Gotuzzo E, Muñoz J. The laboratory diagnosis and follow-up of strongyloidiasis: a systematic review. PLoS Negl Trop Dis. 2013; 7: e2002 10.1371/journal.pntd.0002002 23350004 PMC3547839

[22] Bon B, Houze S, Talabani H, Magne D, Belkadi G, Develoux M et al Evaluation of a rapid enzyme-linked immunosorbent assay for diagnosis of strongyloidiasis. J Clin Microbiol. 2010; 48: 1716–1719. 10.1128/JCM.02364-09 20335415 PMC2863909

[23] Kobayashi J, Sato Y, Toma H, Takara M, Shiroma Y. Application of enzyme immunoassay for postchemotherapy evaluation of human strongyloidiasis. Diagn Microbiol Infect Dis. 1994; 18: 19–23. 8026153 10.1016/0732-8893(94)90129-5

[24] Page WA, Dempsey K, McCarthy JS. Utility of serological follow-up of chronic strongyloidiasis after anthelminthic chemotherapy. Trans R Soc Trop Med Hyg. 2006; 100: 1056–1062. 10.1016/j.trstmh.2005.12.006 16551471

[25] Buonfrate D, Sequi M, Mejia R, Cimino RO, Krolewiecki AJ, Albonico M, Degani M, Tais S, Angheben A, Requena-Méndez A, Muñoz J, Nutman TB, Bisoffi Z. Accuracy of five serologic tests for the follow up of Strongyloides stercoralis infection. PLoS Negl Trop Dis. 2015; 9: e3491. 10.1371/journal.pntd.0003491 PMC4323101 25668740

[26] Bisoffi Z, Buonfrate D, Sequi M, Mejia R, Cimino RO, Krolewiecki AJ et al Diagnostic accuracy of five serologic tests for Strongyloides stercoralis infection. PLoS Negl Trop Dis. 2014; 8: e2640 10.1371/journal.pntd.0002640 24427320 PMC3890421

[27] Suputtamongkol Y, Premasathian N, Bhumimuang K, Waywa D, Nilganuwong S, Karuphong E et al Efficacy and safety of single and double doses of Ivermectin versus 7-day high dose Albendazole for chronic strongyloidiasis. PLoS Negl Trop Dis. 2011; 5: e1044 10.1371/journal.pntd.0001044 21572981 PMC3091835

[28] Datry A, Hilmarsdottir I, Mayorga-Sagastume R, Lyagoubi M, Gaxotte P, Biligui S et al Treatment of *Strongyloides stercoralis* infection with ivermectin compared with albendazole: results of an open study of 60 cases. Trans R Soc Trop Med Hyg. 1994; 88: 344–345. 10.1016/0035-9203(94)90110-4 7974685

[29] Marti H, Haji HJ, Savioli L, Chwaya HM, Mgeni AF, Ameir JS et al A comparative trial of a single-dose ivermectin versus three days of albendazole for treatment of *Strongyloides stercoralis* and other soil-transmitted helminth infections in children. Am J Trop Med Hyg. 1996; 55: 477–481. 8940976 10.4269/ajtmh.1996.55.477

[30] Toma H, Sato Y, Shiroma Y, Kobayashi J, Shimabukuro I, Takara M. Comparative studies on the efficacy of three anthelminthics on treatment of human strongyloidiasis in Okinawa, Japan. Southeast Asian J Trop Med Public Health. 2000; 31: 147–151. 11023084

[31] Nontasut P, Muennoo C, Sanguankiat S, Fongsri S, Vichit A. Prevalence of *Strongyloides* in Northern Thailand and treatment with ivermectin vs albendazole. Southeast Asian J Trop Med Public Health. 2005; 36: 442–444. 15916052

[32] Suputtamongkol Y, Kungpanichkul N, Silpasakorn S, Beeching NJ. Efficacy and safety of a single-dose veterinary preparation of ivermectin versus 7-day high-dose albendazole for chronic strongyloidiasis. Int J Antimicrob Agents. 2008; 31: 46–49. 10.1016/j.ijantimicag.2007.08.014 18023151

[33] Henriquez-Camacho C, Gotuzzo E, Echevarria J, White AC Jr, Terashima A, Samalvides F et al Ivermectin versus albendazole or thiabendazole for Strongyloides stercoralis infection. Cochrane Database Syst Rev. 2016; 1: CD007745. 10.1002/14651858.CD007745.pub3 PMC4916931 26778150

[34] Repetto SA, Ruybal P, Batalla E, López C, Fridman V, Sierra M et al Strongyloidiasis outside endemic areas: long-term parasitological and clinical follow-up after ivermectin treatment. Clin Infect Dis. 2018; 66: 1558–1565. 10.1093/cid/cix1069 29360939

[35] Pérez-Molina JA, López-Polín A, Treviño B, Molina I, Goikoetxea J, Díaz-Menéndez M et al 6-year review of +Redivi: a prospective registry of imported infectious diseases in Spain. J Travel Med. 2017; 24: 1–7. 10.1093/jtm/tax035 28931128

[36] Requena-Méndez A, Buonfrate D, Gomez-Junyent J, Zammarchi L, Bisoffi Z, Muñoz J. Evidence-based guidelines for screening and management of strongyloidiasis in non-endemic countries. Am J Trop Med Hyg. 2017; 97: 645–652. 10.4269/ajtmh.16-0923 28749768 PMC5590585

