# Supplementary material for: Imported strongyloidiasis: Data from 1245 cases registered in the +REDIVI Spanish Collaborative Network (2009-2017)
Source: PLoS Negl Trop Dis. 2019 May 16;13(5):e0007399. doi: 10.1371/journal.pntd.0007399 (PMC6541302; doi:10.1371/journal.pntd.0007399)
Supplement: S1 Table — (DOC) [file pntd.0007399.s002.doc]

Supplementary Table S1. Other infectious diseases diagnosed in patients with strongyloidiasis in +REDIVI (2009-2017).

| **Other infectious diseases** | **Number of patients (n=1245)** |
| --- | --- |
| Chagas disease  Latent tuberculosis infection  Schistosomiasis  HIV infection  Syphilis  HBV infection  Visceral larva migrans  Hookworms  *Giardia lamblia*  Taeniasis  Malaria  Filarial infection  *Trichuris trichiura*  *Entamoeba histolytica*  Arboviral diseases  *Blastocystis hominis*  Genitourinary infections  Tuberculosis  *Ascaris lumbricoides*  *Dientamoeba fragilis*  Bacterial sepsis  Bacterial pneumonia  Hydatid cyst  HCV infection  Cutaneous larva migrans  *Enterobius vermicularis*  Other | 272 (21.8%)  75 (6%)  63 (5.1%)  55 (4.4%)  43 (3.5%)  37 (3%)  32 (2.6%)  25 (2%)  24 (1.9%)  20 (1.6%)  18 (1.4%)  18 (1.4%)  18 (1.4%)  16 (1.3%)  13 (1%)  10 (0.8%)  9 (0.7%)  9 (0.7%)  6 (0.5%)  4 (0.3%)  4 (0.3%)  4 (0.3%)  3 (0.2%)  3 (0.2%)  3 (0.2%)  3 (0.2%)  17 (1.4%) |

**NOTE.** Data are reported as number (%) of patients.
