# Supplementary material for: Imported strongyloidiasis: Data from 1245 cases registered in the +REDIVI Spanish Collaborative Network (2009-2017)
Source: PLoS Negl Trop Dis. 2019 May 16;13(5):e0007399. doi: 10.1371/journal.pntd.0007399 (PMC6541302; doi:10.1371/journal.pntd.0007399)
Supplement: S2 Table — (DOC) [file pntd.0007399.s003.doc]

Supplementary Table S2. Epidemiological characteristics of patients with strongyloidiasis in +REDIVI (2009-2017).

| **Immigrants (n=833)** | | **VFR-Immigrants (n=338)** | | **Travelers (n=74)** | |
| --- | --- | --- | --- | --- | --- |
| Time of residence in Spain, years  Geographical area of origin  South America  Sub-Saharan Africa  Central America/Caribbean  Asia  North Africa  Europe  Top 5 country of origin  Bolivia  Ecuador  Equatorial Guinea  Colombia  Paraguay | 6.8 (SD 5.4)  566 (67.9%)  191 (22.9%)  38 (4.6%)  29 (3.5%)  5 (0.6%)  4 (0.5%)  346 (41.5%)  96 (11.5%)  71 (8.5%)  47 (5.6%)  38 (4.6%) | Duration of the trip, days  Geographical area of origin  South America  Sub-Saharan Africa  Asia  Central America/Caribbean  North Africa  Top 5 country of origin  Bolivia  Ecuador  Equatorial Guinea  Colombia  Philippines  Pre-travel advice  High risk travel | 30 (IQR 30-60)  217 (64.2%)  65 (19.2%)  35 (10.4%)  17 (5%)  4 (1.2%)  108 (32%)  60 (17.8%)  37 (10.9%)  24 (7.1%)  11 (3.3%)  18 (5.3%)  328 (97%) | Duration of the trip, days  Geographical area of origin  Sub-Saharan Africa  South America  Asia  Central America/Caribbean  North Africa  Top 5 country of origin  Thailand  Bolivia  Ecuador  Cameroon, Nigeria, Pakistan, Dominican Republic, and Senegal  Pre-travel advice  High risk travel | 45 (IQR 19.7-227)  31 (41.9%)  18 (24.3%)  15 (20.3%)  9 (12.2%)  1 (1.3%)  6 (8.1%)  5 (6.8%)  4 (5.4%)  3 (4.1%)  33 (44.6%)  60 (81.1%) |

**NOTE.** Data are reported as number (%) of patients or mean/medians (SD/IQR).
