# Supplementary material for: Imported strongyloidiasis: Data from 1245 cases registered in the +REDIVI Spanish Collaborative Network (2009-2017)
Source: PLoS Negl Trop Dis. 2019 May 16;13(5):e0007399. doi: 10.1371/journal.pntd.0007399 (PMC6541302; doi:10.1371/journal.pntd.0007399)
Supplement: S3 Table — (DOC) [file pntd.0007399.s004.doc]

Supplementary Table S3. Treatment outcome comparison between ivermectin and albendazole in patients with strongyloidiasis in +REDIVI with available extra information (2009-2017)^a^.

| **All treatment duration** | **Ivermectin**  **(n=643)** | **Albendazole**  **(n=54)** | ***P* value** |
| --- | --- | --- | --- |
| Cure  Treatment success (cure + probable cure) | 284 (44.2%)  596 (92.7%) | 14 (25.9%)  49 (90.7%) | 0.009  0.6 |
| **Most accepted treatment duration** | **Ivermectin 1-2 days**  **(n=631)** | **Albendazole 5-7 days**  **(n=49)** | ***P* value** |
| Cure  Treatment success (cure + probable cure) | 279 (44.2%)  585 (92.7%) | 12 (24.5%)  45 (91.8%) | 0.007  0.776 |
| **Most preferable treatment duration** | **Ivermectin 2 days**  **(n=606)** | **Albendazole 7 days**  **(n=32)** | ***P* value** |
| Cure  Treatment success (cure + probable cure) | 267 (44.1%)  563 (92.9%) | 8 (25%)  30 (93.7%) | 0.034  1 |

^a^ Patients were only analyzed when having a known outcome (cure, probable cure and failure) and treated with a single drug.
