# Supplementary material for: Imported strongyloidiasis: Data from 1245 cases registered in the +REDIVI Spanish Collaborative Network (2009-2017)
Source: PLoS Negl Trop Dis. 2019 May 16;13(5):e0007399. doi: 10.1371/journal.pntd.0007399 (PMC6541302; doi:10.1371/journal.pntd.0007399)
Supplement: S1 Appendix — (DOC) [file pntd.0007399.s005.doc]

**+REDIVI STUDY GROUP:**

Paloma Aguilera (Centro de Atención Primaria Guayaba, Comunidad de Madrid); María Martínez Serrano (Complejo Hospitalario Universitario de Albacete, Comunidad de Castilla-La Mancha); Magdalena García Rodríguez (Consorcio Hospital General Universitario de Valencia, Comunidad Valenciana ); Marta Díaz Menendez (Hospital Carlos III-La Paz, Comunidad de Madrid); Talía Sainz (Hospital Universitario Infantil La Paz, Comunidad de Madrid); Yolanda Meije, Joaquim Martínez-Montauti, Xavier Sanz (Hospital de Barcelona, Cataluña ); Isabel Pacheco Tenza, Inmaculada Gonzalez Cuello, Belén Martínez López, Jara LLenas (Hospital de la Vega Baja de Orihuela, Comunidad Valenciana ); Mar Masiá, Sergio Padilla (Hospital General de Elche, Comunidad Valenciana); Mónica Romero, Philip Wilkman-Jorgensen (Hospital General de Elda-Virgen de la Salud, Comunidad Valenciana ); José Manuel Ramos Rincón (Hospital General Universitario de Alicante, Comunidad Valenciana ); Eduardo Malmierca; Ana Perez-Ayala, Manuel Lizasoain, Pablo Rojo, Mariano Matarranz, Carlos Zarco (Hospital Universitario 12 de Octubre, Madrid Comunidad de Madrid); Jonathan Fernández Suárez, Jose Antonio Boga Ribeiro (Hospital Universitario Central de Asturias, Oviedo Principado de Asturias); José Manuel Ruiz Giardin, Juan Victor Sanmartín López (Hospital Universitario de Fuenlabrada, Comunidad de Madrid); Eva Calabuig Muñoz (Hospital Universitario La Fe de Valencia, Comunidad Valenciana); Ana Mena Ribas, María Peñaranda Vera (Hospital Universitario Son Espases, Islas Baleares); Daniel Molina-Morant (Hospital Universitario Vall d’Hebron, Cataluña ); Ángel Dominguez, (Hospital Universitario Virgen de la Macarena de Sevilla, Andalucía); Francesca Norman, Begoña Monge, Cesar Henríquez-Camacho (Servicio de Enfermedades Infecciosas, Hospital Ramón y Cajal, Comunidad de Madrid); Antonio Soriano-Arandes, Diana Pou Ciruelo, Cristina Bocanegra, Inés Oliveira (Unitat de Salut Internacional Drassanes, PROSICS, Cataluña).
